# Metadata Correction: Internet Search and Krokodil in the Russian Federation: An Infoveillance Study

**DOI:** 10.2196/jmir.4431

**Published:** 2015-03-20

**Authors:** Andrey Zheluk, Casey Quinn, Peter Meylakhs

**Affiliations:** ^1^ Menzies Centre for Health Policy University of Sydney Sydney Australia; ^2^ PRMA Consulting Ltd, Fleet Hampshire United Kingdom; ^3^ Laboratory for Internet Studies National Research University, Higher School of Economics St Petersburg Russian Federation

The authors of “Internet Search and Krokodil in the Russian Federation: An Infoveillance Study” (http://www.jmir.org/2014/9/e212/) have overlooked an error in the metadata section during the submission and proofreading process. The author Peter Meylakhs carried the affiliation "Internet Studies Lab, Higher School of Economics, St Petersburg, Russian Federation", which should have been "Laboratory for Internet Studies, National Research University Higher School of Economics, St Petersburg, Russian Federation". This error has been corrected in the online version of the paper on the JMIR website on March 20, 2015, together with publishing this correction notice. A correction notice has been sent to PubMed and the correct full-text has been resubmitted to Pubmed Central and other full-text repositories.

